# Extravasation from a Misplaced Intraosseous Catheter

**DOI:** 10.5811/cpcem.2019.4.42561

**Published:** 2019-05-29

**Authors:** Christopher S. Sampson

**Affiliations:** University of Missouri School of Medicine, Department of Emergency Medicine, Columbia, Missouri

## Abstract

A 75-year old female presented in cardiac arrest with a right tibial intraosseous (IO) catheter through which prehospital medications were administered. The catheter, which had been placed by emergency medical services, was noted in the emergency department to be misplaced and was removed. Due to extravasation of the medications, the patient suffered localized tissue necrosis and eventually required skin grafting. This case illustrates the importance of confirming appropriate IO placement.

## CASE PRESENTATION

**Image 1:**
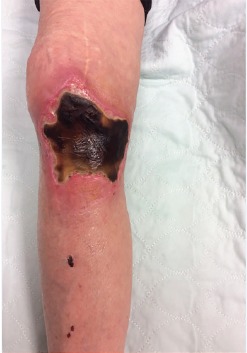
Intraosseous (IO) site, prior to debridement, three weeks after incorrect placement of IO catheter.

**Image 2:**
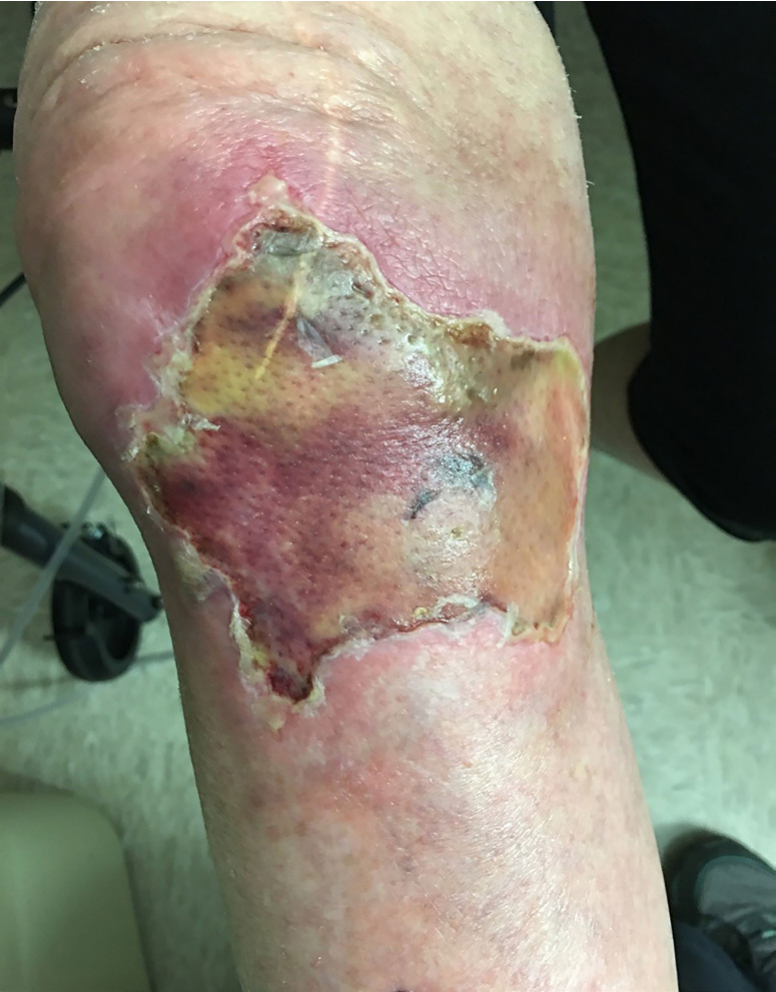
Intraosseous site following debridement.

A 75-year-old female arrived to the emergency department (ED) via emergency medical services (EMS) with a chief complaint of cardiac arrest. The patient had been shopping at a local store and collapsed. Bystanders started cardiopulmonary resuscitation. On EMS arrival her rhythm was found to be ventricular fibrillation. Multiple defibrillations were attempted with return of spontaneous circulation by EMS. During resuscitation an intraosseous (IO) catheter was placed in the right proximal tibia using an EZ-IO (Arrow) device prior to ED arrival. Medications given through the IO catheter by EMS included epinephrine, magnesium, amiodarone, and calcium chloride. On ED arrival, the IO catheter was found to not flush; it was deemed not functional, and new intravenous access was obtained. The IO catheter was removed. On hospital day three, ecchymosis was noted at the IO site. The patient was discharged from the hospital four days after admission and presented to the wound clinic three weeks later (Images 1 and 2).

**Image 3:**
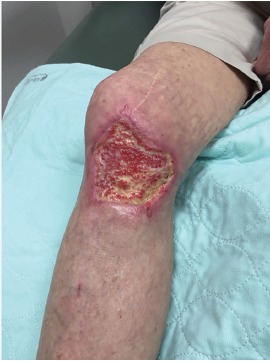
Intraosseous site two months after placement.

After close follow-up (Image 3), skin grafting was performed on the patient’s right leg at three months post event with good healing obtained.

## DISCUSSION

Extravasation of IO catheter infusions is a serious complication due to possible development of tissue necrosis or compartment syndrome. Complications reports are rare but serious and include tissue necrosis, osteomyelitis, fracture, and compartment syndrome.^1^ Emergency providers must ensure proper technique is used for placement, as well as appropriate location. The anteromedial surface of the proximal tibia is the preferred location. After needle placement, aspiration of blood or marrow helps to confirm placement.^2^

**CPC-EM Capsule**

What do we already know about this clinical entity?

Intraosseus (IO) access is a common method used to quickly obtain vascular access when traditional methods fail.

What is the major impact of the image(s)?

Misplaced IO catheters can lead to devastating consequences that can cause long-term morbidity.

How might this improve emergency medicine practice?

The emergency provider should confirm placement of an IO catheter and stay vigilant for signs of extravasation.
